# A novel linkage map of sugarcane with evidence for clustering of retrotransposon-based markers

**DOI:** 10.1186/1471-2156-13-51

**Published:** 2012-06-28

**Authors:** Alessandra C Palhares, Taislene B Rodrigues-Morais, Marie-Anne Van Sluys, Douglas S Domingues, Walter Maccheroni, Hamilton Jordão, Anete P Souza, Thiago G Marconi, Marcelo Mollinari, Rodrigo Gazaffi, Antonio Augusto F Garcia, Maria Lucia Carneiro Vieira

**Affiliations:** 1 Departamento de Genética, Escola Superior de Agricultura “Luiz de Queiroz”, Universidade de São Paulo, 13418-900, Piracicaba, Brazil; 2 Departamento de Botânica, Instituto de Biociências, Universidade de São Paulo, 05508-090, São Paulo, Brazil; 3 Present address: Laboratório de Biotecnologia Vegetal, Instituto Agronômico do Paraná, 86047-902, Londrina, Brazil; 4 CanaVialis/Monsanto Co, Condomínio Techno Park, 13069-380, Campinas, Brazil; 5 Present address: Companhia Vale do Rio Doce, 20020-900, Rio de Janeiro, Brazil; 6 Centro de Biologia Molecular e Engenharia Genética, Universidade Estadual de Campinas, 13083-875, Campinas, Brazil

**Keywords:** *Saccharum* spp, AFLP, EST-SSR, Retrotransposon-based markers, Single-dose markers, Integrated genetic map, Marker distribution

## Abstract

**Background:**

The development of sugarcane as a sustainable crop has unlimited applications. The crop is one of the most economically viable for renewable energy production, and CO_2_ balance. Linkage maps are valuable tools for understanding genetic and genomic organization, particularly in sugarcane due to its complex polyploid genome of multispecific origins. The overall objective of our study was to construct a novel sugarcane linkage map, compiling AFLP and EST-SSR markers, and to generate data on the distribution of markers anchored to sequences of *scIvana_1*, a complete sugarcane transposable element, and member of the *Copia* superfamily.

**Results:**

The mapping population parents (‘IAC66-6’ and ‘TUC71-7’) contributed equally to polymorphisms, independent of marker type, and generated markers that were distributed into nearly the same number of co-segregation groups (or CGs). Bi-parentally inherited alleles provided the integration of 19 CGs. The marker number per CG ranged from two to 39. The total map length was 4,843.19 cM, with a marker density of 8.87 cM. Markers were assembled into 92 CGs that ranged in length from 1.14 to 404.72 cM, with an estimated average length of 52.64 cM. The greatest distance between two adjacent markers was 48.25 cM. The *scIvana_1*-based markers (56) were positioned on 21 CGs, but were not regularly distributed. Interestingly, the distance between adjacent *scIvana_1*-based markers was less than 5 cM, and was observed on five CGs, suggesting a clustered organization.

**Conclusions:**

Results indicated the use of a NBS-profiling technique was efficient to develop retrotransposon-based markers in sugarcane. The simultaneous maximum-likelihood estimates of linkage and linkage phase based strategies confirmed the suitability of its approach to estimate linkage, and construct the linkage map. Interestingly, using our genetic data it was possible to calculate the number of retrotransposon *scIvana_1* (~60) copies in the sugarcane genome, confirming previously reported molecular results. In addition, this research possibly will have indirect implications in crop economics e.g., productivity enhancement via QTL studies, as the mapping population parents differ in response to an important fungal disease.

## Background

Sugarcane is a crop of unquestionable importance for tropical and subtropical regions of the world, where it occupied 24 million hectares in 2009 [1]. Sugarcane is a cost effective renewable resource, with alternative production in food, feed, fiber, and energy e.g. sugar, animal feeds, alcohols, and fertilizers. Brazil is one of the greatest producers and exporters of sugar and ethanol from sugarcane, where cane production reached approximately 625 million tons in 2010, and the sugarcane industry generated a gross annual income of approximately US$ 23 billion [2].

Sugarcane exhibits the most complex genome of any genetically bred crop. Selection based on scientific approaches began in 1888; the first hybridizations were conducted in Java and Barbados, between *Saccharum officinarum* and *S. spontaneum*. *S. officinarum*, known as ‘noble’ canes, are high in sucrose with juicy, thick stalks; low in fiber content, and susceptible to several diseases [3]. *S. spontaneum* is low in sucrose content, but robust, and resistant to abiotic stresses and pests [4]. One hundred years of interspecific hybrid backcrossings with *S. officinarum* (used as the maternal parent), a process called ‘nobilization’, lead breeders to obtain more productive varieties, with ratooning ability, and increased resistance to biotic and abiotic stresses [5,6]. Subsequently, all modern sugarcanes derive largely from intercrossing these canes, followed by intensive selection [7,8]. Therefore, currently grown cultivars are denoted *Saccharum* spp. Varieties are evaluated for rusticity, pest resistance, and high sugar yield prior to release, which requires 12 to 15 years.

The contemporary sugarcane cultivars have a large (10 Gb) and complex genome structure that is highly polyploid, aneuploid (2n = 100 to 130), and have multispecific origins [9] with a complete set of homo(eo)logous genes ranging from 8 to 10 alleles. Classic cytological works as well as fluorescent *in situ* hybridization determined that *S. officinarum* is an octaploid species (2n = 8*x* = 80) that experienced few aneuploid events, and the ploidy level of *S. spontaneum* varies from 5 to 16 (2n = 40 to 128) [10,11]. Genome *in situ* hybridization assays reveal that the ‘R570’ cultivar (2n = 115) shares 80% of its chromosomes with *S. officinarum*, 10% with *S. spontaneum*, and 10% are recombinant chromosomes [12]. These results clearly indicate that two chromosome sets coexist in the sugarcane genome. Vegetative propagation resulted in sugarcane clones with variable chromosome numbers cultivated in field plantations, and numerical and structural alterations continued to accumulate.

Linkage maps are valuable tools to elucidate genetic and genomic organization, particularly in sugarcane due to its increased ploidy levels. However, high inbreeding depression caused by endogamy limits the production of experimental mapping populations, such as F_2_, BCs, RILs, and DH lines. The *S. spontaneum* ‘SES 208’ (2n = 64) linkage map published by Al-Janabi *et al.*[13] was the first map constructed directly from a complex polyploid species, based on single-dose markers (or SDMs) proposed by Wu *et al.*[14], which considers the use of simplex (single allele copy from one parent) to obtain the genetic map. Al-Janabi *et al.*[13] used progeny from a cross between ‘SES 208’, and a diploidized haploid derived from anther cultured ‘SES 208’ and ‘ADP 85-0068’ to estimate linkage. This strategy facilitated direct meiotic analysis in ‘SES 208’, and gametic segregation ratios to be observed. Results showed autopolyploid chromosomal behavior in ‘SES 208’, and the proportion of SDMs to higher dose markers (multiple alleles) fit the assumption of auto-octaploidy, with the absence of repulsion phase linkages. Subsequently, da Silva *et al.*[15] integrated the map of Al-Janabi *et al.*[13] with the simplex-based map of Sobral *et al.*[16]. SDMs linkage relationships were determined using MapMaker software [17].

Later, Grivet *et al.*[18] used selfed progeny to estimate linkage for the elite cultivar ‘R570’. To date, self-fertilized sugarcane progeny are used to map simplex and duplex markers on co-segregation groups (CGs); for instance, Andru *et al.*[19] using the software JoinMap 3.0 [20] constructed a map for the cultivar Louisiana ‘LCP 85-384’. Alternatively, crossing unrelated heterozygous genotypes generates a segregating sibling population (F_1_), which can be valuable for constructing outcrossing species maps, including mapping in sugarcane. From the genetic configurations expected in a segregating F_1_, and denoting gel band presence as *A*, and its absence as *O*, we can make use of the designation *AO xOO* (in a diploid species), or Simplex *x* Nuliplex (in sugarcane) to construct individual maps, one for each parent. This approach, known as the pseudo testcross strategy [21] uses two sets of dominant markers that segregate in a 1:1 ratio. It was applied to estimate linkage in interspecific crosses, where *S. officinarum* was the female (‘Green German’, ‘IJ 76-514’, ‘La Striped’), and *S. spontaneum* the male parent (‘IND 81-146’ and ‘SES 147B’) [22-26]. The Australian cultivars ‘MQ77-340’, ‘Q165’, and ‘MQ76-53’ were mapped similarly [27-29]. Each of these studies determined a comparable number of linkage groups (LGs) and map lengths; e.g. ‘La Striped’ (2n = 80): 49 LGs, 1,732 cM; ‘SES 147B’ (2n = 64): 45 LGs, 1,491 cM [26].

Garcia *et al.*[30] constructed a single integrated genetic map based on simultaneous maximum-likelihood linkage estimates and linkage phase methodology [31], based on a population derived from a cross between two pre-commercial sugarcane cultivars (‘SP80-180’ *x* ‘SP80-4966’). A total of 1,118 single-dose markers were identified; 39% were derived from a testcross configuration between parents segregating in a 1:1 ratio, and 61% segregated in 3:1 ratio, representing heterozygous loci in both parentals with identical genotypes. The final map was comprised of 357 linked markers, including RFLPs, SSRs, and AFLPs assigned to 131 CGs, with a LOD score of 5.0, recombination fraction of 37.5 cM. Authors indicated the simultaneous maximum-likelihood estimates of linkage, and linkage phases were appropriate to generate an integrated genetic map of sugarcane [30]. Then, to enhance existing map resolution, and identify putative functional polymorphic gene loci, Oliveira *et al.*[32] screened EST-SSRs and EST-RFLPs in the same mapping population. Markers analyzed in the previous map were added to 2,303 newly generated polymorphic markers, including 1,669 (72.5%) SDMs; 664 (40%) were scattered on 192 CGs, with a total estimated length of 6,261 cM.

The current development of expressed sequence-based markers such as EST-SSRs, genic SNPs, and TRAPs enrich the genetic data that comprise linkage maps. In sugarcane, due to the necessity of mapping a large number of markers to guarantee a reasonable coverage of its genome with many chromosomes [33], a novel and potentially useful approach is to compile anonymous and putative functional markers. Earlier, the Brazilian Sugarcane Expressed Sequence Tag (or SUCEST) project [34,35] generated 237,954 high-quality ESTs organized into 43,141 putative unique sugarcane transcripts referred to as sugarcane assembled sequences. Based on SUCEST data, Rossi *et al.*[36] developed RFLPs using probes derived from NBS-LRR and LRR conserved domains, and S-T Kinase type resistance genes, and positioned them on the ‘R570’ map. Besides, Rossi *et al.*[37] conducted a transposable element (TE) search, revealing a surprising high number of expressed TE homologues, and found all major transposon families were represented in sugarcane. *Mutator* and *Hopscotch* were later reported as the most represented TE families in the sugarcane transcriptome [38]. The SUCEST database was used to describe two LTR retrotransposon families, which were denoted as *scIvana_1* and *scAle_1*[39]. Both were reported as complete elements, and different members of the *Copia* superfamily. The *scIvana_1* shows low copy numbers (40 to 50) and diversity among copies, and is expressed under specific conditions in low-differentiated tissues; *scAle_1* exhibits high copy numbers in the sugarcane genome (> 1000), is more diversified compared to *scIvana_1*, and active under varied physiological conditions.

Retrotransposon-based markers have been developed using several approaches. In Poaceae species, for example, SSAP was first used to study the distribution of *BARE-1*-like retrotransposable elements in barley genome [40]. In brief, SSAP uses two restriction enzymes to generate a large number of DNA fragments; after that, a retrotransposon-anchored PCR is used to perform a selective amplification. In the 90’s, two new techniques were developed to exploit the polymorphism generated by *BARE-1* genome integration, named REMAP and IRAP [41]. Patterns indicate that although the *BARE-1* family of retrotransposons is dispersed, these elements are clustered or nested locally, and often found near microsatellite sequences. Later, both procedures were reported as useful to screen insertional polymorphisms in populations of *Spartina anglica*, an allopolyploid involved in natural and artificial invasions [42]. These methods are dominant and multiplex, and generate anonymous marker bands. IRAP is based on the PCR amplification of genomic DNA fragments which lie between two retrotransposon insertion sites, and REMAP is based on the amplification of fragments which lie between a retrotransposon insertion site and a microsatellite site. Polymorphism is detected by the presence or absence of the PCR product in both techniques. Lack of amplification indicates the absence of the retrotransposon at the particular locus. In contrast, the RBIP [43] and ISBP [44] score individual loci and are used to search for insertional polymorphisms. RBIP, for example, was used to address the issue of evolution of rice varieties [45] and ISBP was used to analyze diversity in wheat [46]. The RBIP method exploits knowledge of the sequence flanking a TE to design the primers while the ISBP method uses one primer in the element and the other in the flanking DNA sequence.

The overall objective of our study was to generate data on *scIvana_1* and *scAle_1*-based marker distribution to a novel sugarcane linkage map based on a compilation of AFLPs and EST-SSRs.

## Results

### Genotyping and segregation analyses

Excellent AFLPs, EST-SSRs, and *scIvana_1*-based marker banding profiles were obtained [see Additional file 1], despite the size and complexity of the sugarcane genome. The 72 enzyme-selective primer combinations tested provided a range of AFLP band numbers per gel (44 to 174), and polymorphic loci (four to 33). Subsequently, 22 combinations were selected as optimal for genotyping the F_1_ population [Additional file 2]. The combinations generated 102 to 172 AFLP bands per gel, and 19 to 48 polymorphic loci, which revealed 22.1% (685/3,094) segregating loci. Among the segregating loci, 71.2% (488/685) segregated in only one parent, and 28.8% (197/685) segregated in both parents (Table 1). The ‘IAC66-6’ clone and ‘TUC71-7’ variety contributed a respective 52% (254/488) and 48% (234/488) of the loci that segregated in only one parent. The average number of amplified bands and segregating loci per enzyme-primer combination were 140.6 (3,094/22) and 31.1 (685/22), respectively.

**Table 1:** Marker polymorphisms used for mapping, and distribution of the different markers according to the cross type (D1, D2 and C)

**Marker type**	**AFLP**	**EST-SSR**	** *scIvana_1* **	**Total**
Number of scorable bands (evaluated in total)^a^	3,094	273	357	3,724
Number of segregating markers (genotyped)	685	220	87	992
Number of polymorphic markers between parents	488	151	74	713
Number of monomorphic markers between parents	197	69	13	279
Number of single dose markers (SDMs)^b^	535	130	65	730
SDMs of origin from ‘IAC66-6′ [D1]^c^	197	41	23	261
SDMs of origin from ‘TUC71-7′ [D2]^c^	192	60	32	284
SDMs of origin from both parents [C]^d^	146	29	10	185
Total number of linked markers on the map	395	95	56	546
Number of linked markers of origin from ‘IAC66-6′ [D1]	154	33	21	208
Number of linked markers of origin from ‘TUC71-7′ [D2]	164	46	30	240
Number of linked markers of origin from both parents [C]	77	16	5	98

^a^ AFLPs generated from 22 enzyme-selective primer combinations, EST-SSR alleles generated from 41 loci and *scIvana_1*-based markers generated from 6 restriction enzyme-primer combinations.

^b^ Data obtained after Bonferroni’s correction.

^c^ Markers present in only one parent with a 1:1 segregation ratio in the mapping population.

^d^ Markers present in both parents with a 3:1 segregation ratio in the mapping population.

From the 184 EST-SSR loci initially investigated, 22.3% (41) were selected for genotyping [Additional file 2]. These loci revealed 273 alleles with an average of 6.7 alleles per locus; 80.6% (220) segregated in the F_1_ population, 68.6% (151/220) segregating in only one parent, and 31.4% (69/220) in both parents (Table 1). The ‘IAC66-6’ clone and ‘TUC71-7’ variety contributed a respective 43% (65/151) and 57% (86/151) of the alleles that segregated in only one parent.

Among the 16 restriction enzyme-primer combinations used to amplify the retrotransposon *scIvana_1*-based markers, six were selected for genotyping the F_1_ population. These combinations revealed 357 loci; 24.4% (87) behaved as segregating loci. Among them, 85.1% (74/87) and 14.9% (13/87) segregated in only one parent and in both parents, respectively (Table 1). The average number of amplified bands and segregating loci per enzyme-primer combination were 59.5 (357/6) and 14.5 (87/6), respectively [Additional file 2]. The male and female parents contributed a respective 44.6% (33/74) and 55.4% (41/74) of loci that segregated in only one parent. Sixteen retrotransposon *scAle_1* combinations revealed gel profiles; however, amplicon absence, or amplifications associated with non-specific polymorphism prevented profile use in genotyping.

AFLP and *scIvana_1*-based loci exhibited similar levels of segregating alleles in the mapping population (~25%). Notably, both techniques reveal polymorphisms at restriction enzyme cleavage sites. On the other hand, EST-SSR loci showed high levels of segregating alleles in the mapping population (~80%). The polymorphisms observed at EST-SSR loci are due to differences in the size of multiple alleles; we cannot predict if any allele will be fixed in a sugarcane cultivar, which is expected to be highly heterozygous. Both parents contributed equally to polymorphisms i.e. 49.4% ± 3.7% and 50.6% ± 3.8% of the amplicons derived from the male (‘IAC66-6’) and female (‘TUC71-7’) parent, respectively, independent of marker type.

It is important to clarify that we organized our segregation data assuming that homo(eo)logous chromosomes paired faithfully during meiosis, leading to regular bivalent formation as well as normal gametes. It is imperative to emphasize that sugarcane is an artificial genome, highly polyploid, aneuploid, and has interspecific origins, which impedes our capacity to designate co-dominant markers at any locus. Consequently, loci were divided into heterozygous in one parent and null in the other (simplex *x* nuliplex), and heterozygous in both parents (simplex *x* simplex). Based on this model, all markers were scored as dominant (or binary), and were assigned to the expected segregation ratios i.e. 1:1 and 3:1 (Table 1).

A total of 992 segregating loci were genotyped in the mapping population; 685 AFLPs (generated from 22 enzyme-selective primer combinations), 220 EST-SSRs (derived from 41 loci), and 87 *scIvana_1*-based loci (obtained from six enzyme-primer combinations). The expected segregation ratios at each locus (992) were checked using Chi-square tests, adjusting for multiple tests using Bonferroni correction. Then, 730 (73.6%) loci were safely used to build the map, being 535 AFLPs, 130 EST-SSRs, and 65 *scIvana_1*-based loci. The global level of significance used to determine the validity of the segregation ratios of 1:1 and 3:1 was 5.04e-05 (alpha = 0.05/992).

### The genetic map and marker distribution within co-segregation groups

**Figure 1:**
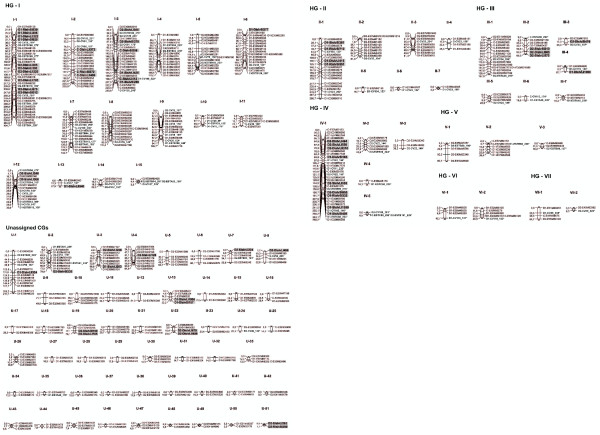
**Linkage map of the population ‘IAC66-6’ *x* ‘TUC71-7′.** Genetic distances between adjacent markers are shown on the left of each co-segregation group (CG). AFLPs constitute the map scaffold; EST-SSR loci appear in bold with asterisk symbols, and *scIvana_1*-based markers are depicted in bold in a gray box. The final map was constructed with 546 markers associated with 92 CGs (in Arabic numerals). Forty-one CGs (42.7%) were assembled into seven putative independent HGs (in Roman numerals). Other CGs (51) were denoted as unassigned groups (U). Note the clustered tendency of some *scIvana_1*-based markers.

The marker number used to perform linkage analyses was 730; 261 were derived from ‘IAC66-6’ (here indicated as D1), 284 from ‘TUC71-7’ (or D2), and 185 were present in both parents (or C). The final sugarcane map was comprised of 546 markers assembled into 92 co-segregation groups (CGs), and 184 markers not assigned to any CG. Coincidently, D1 (208) and D2 (240) markers were distributed into nearly the same number of CGs (51 and 55, respectively). By using the loci that segregated in a 3:1 fashion (98) as bridges, we provided the integration of 19 CGs (I-1, I-2, I-3, I-4, I-6, I-8, I-9, I-12, II-1, II-3, III-1, IV-1, VII-1, U-1, U-2, U-3, U-8, U-13, and U-33). The marker number per CG ranged from two to 39 (Figure 1). The total map length was 4,843.19 cM, with a marker density of 8.87 cM. The CG length covered a range from 1.14 to 404.72 cM, with an estimated average of 52.64 cM. The greatest distance between two adjacent markers was 48.25 cM (CG I-3).

Forty-one CGs (44.6%) were assembled into seven putative independent homo(eo)logous groups (HGs), which were recognized based on 82 alleles generated by 24 EST-SSR loci. The remaining loci (ESTB189, ESTB191, CV11, and CV86) did not contribute to CG assembly; only one allele was positioned on the respective HG. The CG number assembled into HGs ranged from two to 15, the largest (HGI) contained 210 markers, and the smallest (HGVII) six markers, which was clearly a very non-uniform distribution (Figure 1). Eight previously mapped EST-SSR loci [32] were placed here on HGI (ESTA53, ESTB94, ESTB100, and ESTB118), HGIII (ESTA48), HGIV (ESTC80 and ESTB99), and HGV (ESTB60).

The mapped proportion of *scIvana_1*-based loci was 86.2% (56/65), greater than AFLPs (73.8%, 395/535) and EST-SSRs (73.1%, 95/130). The *scIvana*_*1*-based loci (56) were positioned on 21 CGs, which were represented in four HGs. However, there is some evidence that these markers may not be regularly distributed (Figure 1); only one marker was placed in groups I-6, I-13, III-3, III-4, U-1, U-2, U-4, U-7 and U-8, and more than five markers were placed in groups I-1, I-3, and IV-1. The evidence for clustering of *scIvana*_*1*-based markers was verified by the Chi-square goodness-of-fit test that results in a p-value 4.64e-04. This model has been used for testing if marker distribution deviates significantly from a random distribution in genetic maps i.e. segregating as expected in a Poisson distribution [47]. The number of regions with two (6) and three (1) adjacent markers was higher than expected only by chance (2 and 0.09, respectively). Interestingly, distances between some of the adjacent *scIvana*_*1*-based markers were lower than 5 cM, notably in groups, I-1, I-2, III-2, IV-1, and U-51 (Table 2). This value was the same used by Rossi *et al.*[36] to define NBS/LRR RGA clusters.

**Table 2:** Retrotransposon clustering and its position into the sugarcane genetic map

**Cluster**^**a**^	**Markers**	**Cluster size**^**a**^**(cM)**	**Map position**^**b**^	
			**HG**	**CG**
1	D1-RIsIvSI530, D1-DIsIvLI496, D1-DIsIvLII322	6.64	I	I-1
2	D2-DIsIvLI385, D2-SIsIvLI390	1.74	I	I-1
3	C-RIsIvSI372, C-DIsIvLII405	0.61	I	I-2
4	D2-SIsIvSI210, D2-DIsIvLI905	0.7	III	III-2
5	D2-DIsIvLI494, D2-SIsIvLI550, D2-DIsIvLII310	5.66	IV	IV-1
6	D2-SIsIvLI263, D2-RIsIvSI280	1.14	-	U-51

^a^ Defined by the distance between the flanking markers.

^b^ HG, homo(eo)logous groups; CG, co-segregation groups.

We subsequently obtained the total *scIvana_1*-based marker numbers (357), divided by the number of enzyme-primer combinations used to obtain the amplicons (6), and the result was ~60. We propose this as the number of retrotransposon *scIvana_1* copies in the sugarcane genome. Similarly, by obtaining the total number of *scIvana_1*-based markers each parent contributed separately, the following results correspond to the number of *scIvana_1* copies in the respective ‘IAC66-6’ and ‘TUC71-7’ genomes: 53 (316/6) and 54 (324/6).

However, the total number of *scAle_1*-based fragments was 1008. Nine restriction enzyme-primer combinations were used to amplify the *scAle_1*-based fragments, an average of 112 per combination. The results did not facilitate the identification of any clear polymorphisms in the gels. Consequently, the data were not used for mapping studies.

### Molecular validation of *scIvana_1*-based fragments

Twenty-five *scIvana_1*-based markers selected for genotyping were sequenced; and 64% (16/25) showed homology with known nucleotide sequences deposited in GenBank (Table 3). Most (13 sequences) showed homology with *scIvana_1* sequences, and others showed homology to *Zea mays* (DIsIvSI337), *Oryza minuta* (DIsIvSI390, DIsIvSI412), and *Sorghum bicolor* (SIsIvSI180) sequences. The DIsIvLI228 fragment also exhibited homology with a *Saccharum* chloroplast sequence. Six of the sequences were mapped, DIsIvSI163, DIsIvSI208, DIsIvSI160, SIsIvLI240, SIsIvLI412, and DIsIvLI415, and two were tightly linked with EST-SSRs. The DIsIvSI337 fragment was submitted for tblastx search, and revealed similarity with a hypothetical, highly conserved protein of unknown function in *Arabidopsis thaliana*, and other species. Similarly, DIsIvSI390 and DIsIvSI412 fragments were aligned with an *Oryza minuta* sequence; tblastx indicated similarity with a hypothetical protein of unknown function. The SIsIvSI180 fragment exhibited similarity to two *Sorghum* sequences, one to a retrotransposon of *S. bicolor*[48]. Finally, the DIsIvLI228 fragment exhibited a certain identity with a ribosomal protein chloroplast sequence of *Saccharum* ‘SP80-3280’ (Table 3).

**Table 3:** ** *scIvana_1* -based fragments with homology to nucleotide sequences deposited in GenBank (e-value < e**^**-5**^**)**

**Marker code**	**Size (bp)**	**GenBank accession no.**	**Homology**	**E-value**^**a**^
DIsIvSI	138	DQ115032.1	*Saccharum* ‘SP80-3280’ clone SCCCCL6002A07 Tnt1-like, partial sequence	2e-27
DIsIvSI	160	DQ115032.1	*Saccharum* ‘SP80-3280’ clone SCCCCL6002A07 Tnt1-like, partial sequence	8e-27
DIsIvSI	208	DQ115032.1	*Saccharum* ‘SP80-3280’ clone SCCCCL6002A07 Tnt1-like, partial sequence	5e-29
DIsIvSI	310	DQ115032.1	*Saccharum* ‘SP80-3280’ clone SCCCCL6002A07 Tnt1-like, partial sequence	2e-28
DIsIvSI	337	EU969904.1	*Zea mays* clone 337091 mRNA sequence	6e-28
DIsIvSI	390	DQ115032.1	*Saccharum* ‘SP80-3280’ clone SCCCCL6002A07 Tnt1-like, partial	5e-178
			sequence	
		AC216031.1	*Oryza minuta* clone OM__Ba0016E09, complete sequence	1e-70
DIsIvSI	412	DQ115032.1	*Saccharum* ‘SP80-3280’ clone SCCCCL6002A07 Tnt1-like, partial sequence	2e-147
		AC216031.1	*Oryza minuta* clone OM__Ba0016E09, complete sequence	4e-60
SIsIvSI	158	DQ115032.1	*Saccharum* ‘SP80-3280’ clone SCCCCL6002A07 Tnt1-like, partial sequence	2e-28
SIsIvSI	180	AC169373.2	*Sorghum bicolor* clone SB_BBc0188M08, complete sequence	3e-36
		FN431662.1	*Sorghum bicolor* BAC contig 24P17c, cultivar Btx623	1e-34
SIsIvSI	245	DQ115032.1	*Saccharum* ‘SP80-3280’ clone SCCCCL6002A07 Tnt1-like, partial sequence	2e-28
SIsIvSI	330	DQ115032.1	*Saccharum* ‘SP80-3280’ clone SCCCCL6002A07 Tnt1-like, partial sequence	2e-28
RIsIvSI	195	DQ115032.1	*Saccharum* ‘SP80-3280’ clone SCCCCL6002A07 Tnt1-like, partial sequence	5e-29
DIsIvLI	228	AE009947.2	*Saccharum* ‘SP-80-3280’ chloroplast, complete genome	3e-90
DIsIvLI	272	DQ115032.1	*Saccharum* ‘SP80-3280’ clone SCCCCL6002A07 Tnt1-like, partial sequence	9e-106
DIsIvLI	415	DQ115032.1	*Saccharum* ‘SP80-3280’ clone SCCCCL6002A07 Tnt1-like, partial sequence	2e-93
DIsIvLI	605	DQ115032.1	*Saccharum* ‘SP80-3280’ clone SCCCCL6002A07 Tnt1-like, partial sequence	2e-102

^a^ All are standard nucleotide-nucleotide BLASTn scores.

## Discussion

AFLPs have been used to assess genetic diversity in germplasm collections of sugarcane and close relatives [49-52], and to build linkage maps [19,53]. In light of these studies, AFLPs have been informative in generating a substantial amount of unambiguous polymorphic markers. For example, Andru *et al.*[19] reported 64 AFLP restriction enzyme-primer combinations, and detected 816 polymorphic loci; in the present study, 22 combinations revealed 685 polymorphic loci. We suggest the differences were the result of the genotyping population, the first with increased homozygosity (S_1_ progeny) relative to a segregating F_1_ population. In both studies, AFLPs were a viable marker to build a scaffold for other marker types, including expressed sequence-based markers, which could be positioned on the scaffold. Furthermore, this scaffold is particularly important in sugarcane, as expressed sequences are physically too far apart.

From the 22 enzyme-selective primer combinations selected in this study to detect AFLPs, 14 were previously tested to build maps for the ‘R570’ cultivar [53], the *S. officinarum* ‘IJ 76-514’ clone, and ‘Q165’ [28], ‘Q117’ and ‘MQ77-340’ [27] cultivars as well as for the F_1_ population ‘SP80-180’ *x* ‘SP80-4966’ [30]. Based on shared AFLPs and SSRs, the map comparisons of ‘R570’, ‘Q165’, ‘Q117’, and ‘MQ77-340’ cultivars were done [33]; several co-segregation groups (CGs) were aligned, and homo(eo)logous groups (HGs) associated, which received the same designation. As several common alleles were positioned, therefore suitable data should be available to construct a reference map for sugarcane commercial varieties, despite the pedigree complexity. For instance, a comparison between the map built based on the present study and the one published by Pastina *et al.*[54] is possible. Both are integrated linkage maps that share some common SSRs. In both maps, ESTA53, ESTB94, ESTB100 and ESTB118 were assigned to HGI as well as ESTA48 was mapped in the same grouping, HGIII. In addition, HGV contains three CGs that share the ESTB60 locus, which was assigned to HGVII of Pastina’s map [54]. This suggests a possible correspondence between these HGs.

Several authors have applied SSR markers to estimate linkage in sugarcane [19,30,32,33]. Due to the multiallelic nature and relative abundance of SSRs in plant genomes, they have utility to identify HGs in polyploid species [33]. Based on this principle, Rossi *et al.*[36] identified 66 CGs (of 128) assembled into seven HGs from the French cultivar ‘R570’ linkage map. Similarly, Aitken *et al.*[28] identified 136 CGs assembled into eight HGs from the Australian cultivar ‘Q165’ linkage map. Oliveira *et al.*[32] identified 120 CGs (of 192) assembled into 14 HGs from a map of the progeny derived from a single cross between ‘SP80-180’ and ‘SP80-4966’.

Here, we compiled 41 CGs (of 92) into seven putative HGs. Interestingly, six EST-SSR loci were duplicated within chromosomes CV22, CV38, CV78, CV100, ESTB14, and ESTB94, which were positioned on HG I and IV (Figure 1). Duplicated genomic regions were reported to occur in various sugarcane maps [23,28,32,33], and are possibly a consequence of the multispecific origins of the modern cultivars. Structural genomic rearrangements, including the movement of transposable elements (TEs) may also explain the duplications [55].

We used an innovative approach by mapping transposon-based markers in sugarcane using the NBS-profiling technique. In other plant species, TEs have been mapped using SSAP. This marker system was applied in barley (namely the TE *BARE-1*) [40], wheat (TEs, *Wis2A-1 A* and *BARE-1*) [56], lettuce, (*Tls1* and *Tls2*) [57], and tomato (*ToRTL1T265* and *Tnt1*) [58]. Due to the advanced knowledge in tomato genetics, it was possible to determine that polymorphic insertions were primarily located in the centromeric regions. Both the above-mentioned approaches increase the available information regarding retrotransposon distribution over plant linkage groups. The NBS-profiling protocol efficiently targeted *scIvana_1* retrotransposon sequences and, at the same time, produced a polymorphic multilocus marker profile that was enriched for these sequences. Both the SSAP approach and the NBS-profiling technique investigate polymorphic restriction sites and the presence or absence of the retrotransposon sequence. SSAP uses two restriction enzymes (one with frequent cut sites and the other with rare cut sites), generating a large number of DNA fragments before performing the selective amplification. PCR results from the use of a primer that is complementary to the adaptor sequence and other complementary to the retrotransposon sequence. Since we have to choose two enzymes which have no recognition sites for restriction in the retrotransposon sequence, it reduces the number of combinations (enzyme/primer) to be tested. The NBS-profiling technique only uses one restriction enzyme (with frequent or rare cut sites), generating a small number of DNA fragments to be selected and consequently be stained and visualized separately in the gel. This is especially important considering the large genome size of sugarcane. Using enzymes that have no recognition sequences in the retrotransposon *scIvana_1* (combined with primers complementary to its sequence), it was possible to estimate the number of copies of this element in the genome.

Among the 16 restriction enzyme-primer combinations used to amplify *scIvana_1*, the enzymes *Dra*I and *Ssp*I resulted in an increased number of scorable bands and polymorphic loci compared to *Alu*I and *Rsa*I. Earlier studies have shown rare-cutting enzymes such as *Dra*I and *Ssp*I are more suitable for restricting sugarcane DNA due to its large size. However, enzymes that frequently cut sugarcane DNA have the potential to generate an enormous number of fragments, and consequently affect other protocol steps, and subsequent results. All enzyme-primer combinations resulted in non-amplification for *scAle_1*. Primer design was challenging due to sequence diversity among *scAle_1* copies. When amplicons were obtained, all combinations resulted in ∼ 112 bands per gel, which prevented polymorphism identification. Alternatively, a reduction in the number of bands per gel can be reached by adding selective bases at the 3'-end of PCR primers, as previously demonstrated in barley [40]. Queen *et al.*[56] used SSAP to study the elements *Wis2A-1A* and *BARE-1* in wheat, and four selective nucleotides were added as an attempt to reduce the number of amplified fragments. Both elements are known to have 1,000 copies in the wheat genome, and good results were obtained for genotyping and mapping when applying this strategy. Besides this, we should try to produce markers derived from *scAle_1* subfamilies, therefore having an estimate of the number of copies of each subfamily in the sugarcane genome.

The mean number of amplicons obtained by restriction enzyme-primer combinations was very similar to the number estimated by molecular methods; 40 to 50 copies of *scIvana_1* were detected in the sugarcane genome, and *scAle_1* exceeded 1,000 copies [39]. These results were congruent with 56 *scIvana_1*-based loci positioned on the linkage map, exhibiting preferential cluster distribution along 21 CGs. In large-genome cereals, Bennetzen [59] reported retrotransposon distribution as nested insertions in highly heterochromatic transposon clusters. Later, authors reported there appears to be some clustering of TE *BARE-1*/*Wis-2-1 A*-based markers on the linkage map of wheat [56], and transposon cluster interference with recombination machinery acting in adjacent euchromatic regions in maize [60]. When additional genomic data is available for sugarcane, it will be interesting to investigate if genes adjacent to retrotransposon clusters are less recombinogenic. Dooner and He [60] suggested the more condensed chromatin state of retrotransposon clusters in maize might interfere with recombination machinery access in adjacent euchromatic regions. Additionally, in a recent review published by Kalendar *et al.*[61] authors indicated that at least in cereals and citrus retrotransposons are often locally nested one into another and in extensive domains that have been referred to as ‘retrotransposon seas’ surrounding gene islands.

We are possibly facing an association between clustered retrotransposon sequences, the inhibition of DNA recombination, an explanation of the small map distance between adjacent retrotransposon-based markers, and the element copy number in plant the genome. This should explain *scIvana_1* properties, such as low copy numbers (~60) with expression and mobility under strict control, conversely against the properties of *scAle_1* retrotransposons. Therefore, mapping *scAle_1* element is of great interest, as well as the location of these two elements in chromosome regions.

The segregation results presented here independently indicated that AFLPs, EST-SSRs, or *scIvana_1*-based loci were consistent with the outcome of former studies [26,28,32,53,62] i.e. most markers (~70%) were SDMs. Furthermore, a substantial number were unassigned markers, in addition to variation in the marker number per CG.

The genetic map constructed here (‘IAC66-6’ *x* ‘TUC71-7’) has 546 SDMs covering 4,843 cM that were ordered in 92 CGs, with a marker density of 8.87 cM. The genetic map recently published by Pastina *et al.* (‘SP80-180’ *x* ‘SP80-4966’) has 317 markers covering 2,468 cM that were ordered in 96 CGs, with a marker density of 7.5 cM [54]. These are both integrated maps constructed based on segregating F_1_ populations. Note that the number of CGs is somewhat high in Pastina’s map, but it is shorter and denser. Cultivar maps are established using self-fertilized populations, and therefore are not comparable to other maps built based on different backgrounds. For instance, the ‘LCP 85-384’ map has 784 markers covering 5,617 cM that were assigned to 108 CGs, with a marker density of 7.16 cM [19], and ‘Q165’ map has 910 markers covering 9,058 cM that were assigned to 116 CGs, with a marker density of 9.95 cM [28]. Note that, in this case, the shorter and denser map is the one with a low number of CGs.

Enhancement of sugarcane genetic maps should include additional segregation ratios in mapping analyses, and an increased number of informative SNP- and SSR-loci segregating in larger populations. In addition, there is a need for meiotic studies that it is an important component of future studies in deciphering the genetic configuration of sugarcane genotypes.

Finally, it is important to note that the parents of the mapping population differ in response to the *Sporisorium scitamineum* infection; therefore we expect that offspring segregate for this trait. Consequently, the genetic map established here should be used to localize quantitative loci. It will certainly contribute to a better view on the genetic architecture of smut resistance in sugarcane, as little is known on this subject [63,64]. As recently shown in Pastina *et al.*[54] integrated genetic maps are useful for mapping QTLs. Based on interval mapping and mixed models, authors map QTL effects on a segregating progeny from a cross between two pre-commercial cultivars. The same approach should be interesting to be applied using the present map that includes retrotransposon-based markers. Moreover, we should improve McNeil *et al.*’s strategy [65] by aligning marker sequences tightly linked to QTLs for smut resistance with data from the sugarcane genome sequencing project currently underway [66].

## Conclusions

The results of this study showed that AFLPs are a viable marker to create a scaffold for a linkage map, where other marker types can be positioned including expressed sequence-based markers. Results indicated the use of a NBS-profiling technique was efficient to develop retrotransposon-based markers in sugarcane. The simultaneous maximum-likelihood estimates of linkage and linkage phase based strategies confirmed the suitability of its approach to estimate linkage, and construct the linkage map. Interestingly, using our genetic data it was possible to calculate the number of retrotransposon *scIvana_1* (~60) copies in the sugarcane genome, confirming previously reported molecular results. In addition, this research possibly will have indirect implications in crop economics e.g., productivity enhancement via QTL studies, as the mapping population parents differ in response to an important fungal disease.

## Methods

### Plant material and genomic DNA extraction

The mapping population was composed of 188 individuals derived from a single cross between ‘IAC66-6’ and ‘TUC71-7’. The male parent ‘IAC66-6’ is a clone with low sucrose content, large diameter stems, and is susceptible to sugarcane smut, a fungal disease caused by *Sporisorium scitamineum*; the female parent, the Argentinean variety ‘TUC71-7’, exhibits a higher sucrose content, lower diameter stems, and resistance to smut disease. This disease limits the use of recent high-yielding sugarcane varieties developed in Brazil. The cross was made under field conditions at the CanaVialis/Monsanto Company experimental station, located in the northeastern state of Alagoas, Brazil (S 09°39'57''; W 35°44'07''). Sugarcane successfully flowers and sets seed at the locality due to light period duration (photoperiod), and plants were therefore cultivated at this site. Seeds were harvested, germinated in plastic boxes, and transported to the southeastern state of São Paulo (S 22°19'49''; W 47°10'21'') for field cultivation.

DNA was isolated from young leaves of F_1_-progeny and parental plants using the CTAB-based extraction procedure [67], with minor modifications. DNA concentrations were carefully estimated following electrophoresis on ethidium bromide-stained agarose gels using molecular weight standards; aliquots of 50 ng/μl were prepared following quantification.

### Generation of AFLP profiles

AFLPs were amplified based on the protocol described by Vos *et al.*[68] and applied to sugarcane. Briefly, 250 ng of genomic DNA was double digested with 6U of *Eco*RI (Promega) and *Mse*I (NE Biolabs) in a 25-μl reaction mixture (10 mM Tris–acetate pH 7.5, 10 mM Mg-acetate, 50 mM K-acetate, 5 mM DTT, 1 X BSA) for 4 h at 37°C. Restrictions were terminated by heat inactivation for 20 min at 65°C. The resulting fragments were ligated to adapter sequences by addition of an equal volume of ligation solution comprised of 0.25 μM *Eco*RI adapter, 2.5 μM *Mse*I adapter, 1 X enzyme reaction buffer, and 67 U of T4 DNA ligase (400 units/μl, NE Biolabs). Incubations were performed for 16 h at 16°C, and reactions were terminated by heat inactivation. The adapter-ligated DNA (3 μl) was used for pre-selective amplification with primers based on the adapter sequences with one selective nucleotide at the 3’ end (*Eco*RI + A and *Mse*I + C). Pre-selective amplification was performed in a 20 μl reaction mixture containing 1.5 mM MgCl_2_, 0.5 mM each dNTP, 250 nM each primer, 1 X enzyme reaction buffer, and 3 U *Taq* DNA polymerase (Promega). Amplifications were conducted under the following conditions: 94°C for 2 min; 26 cycles of 94°C for 60 s, 56°C for 60 s, 72°C for 60 s; and a final elongation at 72°C for 5 min. For the selective step, 1.5 μl of a 5-fold water diluted pre-selected PCR product was used as DNA template. The 20 μl reaction mixture contained 1.5 mM MgCl_2_, 0.2 mM each dNTP, 250 nM *Eco*RI + ANN, 300 nM oligo *Mse*I + CNN, 1.6 U *Taq* DNA polymerase (Fermentas), and 1 X enzyme reaction buffer. Selective amplification was conducted under the following conditions: 94°C for 2 min; 12 cycles of 94°C for 30 s, 65°C for 30 s, 72°C for 60 s; the final 23 cycles had similar conditions with the exception of a 56°C primer annealing temperature, and a final elongation at 72°C for 2 min. Following PCR, the amplified products were mixed with an equal volume of denaturing buffer containing 95% formamide, 10 mM EDTA (pH 8.0), 0.2% bromophenol blue, and 0.2% xylene cyanol. Samples (3 μL) were loaded into 5% (w/v) polyacrylamide gels (acrylamide/bis-acrylamide, 19:1). Electrophoresis was performed at a constant power of 70 W for 4 h, using a Sequi-Gen® GT (Bio Rad) apparatus. Gels were silver-stained according to the protocol described by Creste *et al.*[69].

Seventy-two different restriction enzyme and selective primer combinations were examined using DNA from both parents in duplicate reactions. Combinations that exhibited good profiles, and revealed a large number of loci and polymorphism rates (≥ 20%) were selected for genotyping the F_1_ population. The polymorphism rate between parents was calculated by assessing the number of bands present in one parent and absent in the other, in relationship to the total number of amplified bands.

### EST-SSRs amplification

In analyzing the SUCEST database, Pinto *et al.*[70] identified 2005 clusters containing SSRs. Primer sets were subsequently developed from these data, and used in polymorphism analyses [70-73]. In addition, Maccheroni *et al.*[74] analyzed 352 and 122 sugarcane ESTs available in both public [75] and private [76] databases to establish sugarcane SSRs. Primer sets were developed from these sequences. In the present study, we used published [72-74], and non-published primer sets developed by CanaVialis/Monsanto.

EST-SSRs amplification was performed in a final volume of 10 μl in 96-well thermocycler plates. Approximately 20 ng of template DNA was mixed in a solution of 0.25 μM of each forward and reverse primer, 0.2 mM each dNTP, 2.0 mM MgCl_2_, 1X Colorless Go *Taq* buffer, and 1.0 U Go *Taq* Flexi DNA Polymerase (Promega). Amplifications were performed using two thermal cycling programs. The first program was conducted under the following conditions: 94°C for 3 min; 31 cycles of 94°C for 60 s; primer annealing at varied temperatures for 60 s; extension at 72°C for 60 s; and a final elongation at 72°C for 2 min. The second was conducted under the following cycle parameters: an initial denaturation step at 94°C for 5 min; followed by 35 cycles of 94°C for 30 s; primer annealing at varied temperatures for 30 s; extension at 72°C for 30 s; and a final elongation at 72°C for 60 min. PCR products were analyzed by two methods, denoted S and F. Amplicons were resolved by 5% (w/v) denaturing polyacrylamide gel electrophoresis, and silver stained (S) as above described; or capillary electrophoresis using a MegaBACE 1000® genotyping system (GE Healthcare Life Sciences). For capillary electrophoresis, forward primers were labeled with fluorescent dyes (F) (fluorophore NED or 6-FAM, Applied Biosystems), and fragments were verified with the Fragment Profiler version 1.2®.

Polymorphisms between parental genotypes were assessed by amplifying 184 EST-SSRs using DNA from ‘IAC66-6', ‘TUC71-7', and a sample from the mapping population (F_1_). The data included 33 EST-SSRs developed by Oliveira *et al.*[72], two from Marconi *et al.*[73], and three from Maccheroni *et al.*[74]. In addition, 146 EST-SSRs were available from CanaVialis/Monsanto (unpublished data). Results showed 41 polymorphic loci, which did segregate in the progeny sample; therefore, these loci were applied to genotype the mapping population. Details on these sugarcane EST-SSRs are presented in Table 4.

**Table 4:** Details on the sugarcane microsatellite loci derived from expressed sequence tags (ESTs)

**Marker code**	**Repeat motif**	**Forward primer (5′**→**3′)**	**Reverse primer (5′**→**3′)**	**PCR**^**e**^	**AT**^**f**^	**D**^**g**^	**Allele number and size range**^**h**^	
ESTA26 ^**a**^	(TG)_11_	GGCAGCCCCACATCTTCCT	GGGCACAAGCATCCGAACC	1	56.0	S	4	172-186
ESTA48 ^**a**^	(CA)_8_	AGCAACTCCGGCCTCTCCTG	CTTTCTGTTTTGCTCCTCCGTCTG	1	62.7	S	10	233-295
ESTA53 ^**a**^	(TG)_8_	TGGAAATGGCAGCTGGTCTCGT	ATGCACGTACCAGAGGGAGATTTG	1	58.9	S	9	168-192
ESTA61 ^**a**^	(AT)_12_	ACCTCAGTCTCCTCCTCAACC	TATACTACACATGCACAGGCTACG	1	56.4	S	5	236-246
ESTB14 ^**a**^	(CGT)_8_	TGAGGGAATGAATGGACTGG	CCACCACCACCATACCTGTC	1	52.0	S	9	285-315
ESTB55 ^**a**^	(CCA)_5_	CTTCTTGGCCTTGGCGTTACTGA	GCTAGCTGGCCCCATTTCCTCT	1	60.0	S	3	118-124
ESTB60 ^**a**^	(TTG)_10_	AGCCGCAATGAATCCAACTG	CTCTAGCTCCGACGATGATACCTC	1	61.0	S	8	157-206
ESTB82 ^**a**^	(CGT)_9_	CGTCGATCGAGATGAAGAAGG	GAAGCAGTCGTGGAAGTGGAG	1	62.7	S	5	245-263
ESTB94 ^**a**^	(CTT)_9_	GAGGCAGCCAGGCAGGTCAC	GGTGGCAGTGTTCAGGCAGATG	1	61.0	S	10	210-279
ESTB99 ^**a**^	(TCG)_5_	GAGGTCCTTCTTGTAGTTGTATGC	GTGCCGGAGGATTTGATG	1	64.7	S	4	215-224
ESTB100 ^**a**^	(TCG)_6_	CCACGGGCGAGGACGAGTA	GGGTCCTTCTTCGCCTCGTG	1	64.7	S	13	240-278
ESTB118 ^**a**^	(TTC)_6_	CTTGGCTAGGGTTTCTTGAGTCGT	CATGGCTTTTGGCTTGCTTCT	1	61.0	S	5	106-163
ESTB189 ^**b**^	(TCA)_10_	GTAAGGAAGAAGCAACAAACAACAG	GATTCGATGCAACTCTCCTGTAAA	1	60.0	S	5	261-280
ESTB191 ^**b**^	(GCT)_5_	GCGCCATCAGGGAAGCCAAAAC	GCGCGTGCGAGCAGATGAAC	1	60.0	S	5	213-226
ESTC80 ^**a**^	(ATTC)_3_	ATTCTTTCTCCCCCTGTTGTGC	GTCGCCAGATCGCTTTCGTT	1	58.9	S	7	188-292
CV06 ^**c**^	(AATT)_13_	TCTCAAGCTTCGCCAGCTA	TGGCTCGGCTGTAGGAATTA	2	60.0	S	3	188-230
CV11 ^**c**^	(GAA)_6_	TGGCATGTGTCATAGCCAAT	CCCCAACTGGGACTTTTACA	2	60.0	S	6	227-242
CV22 ^**c**^	(AGGG)5	CACTACTCGCCCCGATTTC	CGAGTGCTTCTCCATCTGC	2	64.0	F	8	140-166
CV23 ^**c**^	(GGAA)_7_/(AGG)_6_	GAACTGCTCACTGGCTCCTC	GTAGAAGTCCGTCGCCGTAA	2	64.0	F	9	150-206
CV24 ^**c**^	(CCAA)_5_/(CACCT)_4_	TCGGAGAAGTTGACCGAGTT	GGTTTAGAGTTGGGGCCTTC	2	60.0	F	7	187-205
CV29 ^**d**^	(ATCT)_14_	TCGCGTCCACCAATGTAACC	GCGTGCATCGCTTGTGTCTT	2	64.0	F	10	85-133
CV37 ^**d**^	(TTTC)_15_	GGATGGACGACGTGTCCTGG	ATAAAGTGGCCGCTTGGATTGA	2	64.0	F	6	117-155
CV38 ^**d**^	(CTTTT)_18_	GAAGCAGGGGCCTCAAGTTG	GTCAAACAGGCGATCTGGCTC	2	64.0	F	9	109-199
CV46 ^**c**^	(GGTAA)_11_	TGTTCCAAGTTCATGCGCTCC	ATGCATGCAGGTTCAAAAGCAG	2	64.0	F	5	146-188
CV51 ^**c**^	(TGT)_13_	CTACCCCAACTTGCTTGGGAC	GACTGGAACAAAGACGGACTG	2	64.0	F	3	147-160
CV53 ^**c**^	(AAAAT)_5_/(TTTAT)_6_	CCCCACCGTAGCTTGTGCAT	AAACGTGCACATGCTTGTATGC	2	64.0	F	7	160-183
CV58 ^**c**^	(ATAGAT)_10_	CGGGTAGTTAGGAGGAGATGG	GTCATCCATTTTGGAACGAATGG	2	64.0	F	6	153-195
CV78 ^**c**^	(CTGTG)_9_	ACGAGGCCACCATAGAACATG	GCAATTGGGAGGAGAGGAATG	2	64.0	F	9	144-203
CV79 ^**c**^	(CTATAT)_11_/(TATAGA)_6_	GGCACTGCTGGTGGTTGATTG	TCCCACATCAAGAGGCAGCTA	2	64.0	F	7	136-197
CV86 ^**c**^	(AATT)_8_	CCTCAGCAGCCCAAAGTCCT	GTCGGAATCAGCCGGATTAGC	2	64.0	F	5	159-187
CV91 ^**c**^	(GCC)_6_/(GCA)_6_	AAAGGAAATCGCCCTCCGTCT	CCGATGATGAGCCAGCAATCC	2	64.0	F	8	175-197
CV94 ^**c**^	(AAAAAG)_5_/(CGT)_5_	GGCAGGCCAAGATGAATGAAG	AGCACAGCGGAGGGTACGG	2	64.0	F	4	187-205
CV100 ^**c**^	(GAG)_13_	CTGTTGAGGAGCCGGATGAG	CTCTTCCGATGGCTCGGTCT	2	64.0	F	9	222-256
CV101 ^**c**^	(ATC)_23_	GTCGTCGTCGTCACGATCATC	AGTTGACGGCATGGTTCTTGC	2	64.0	F	11	111-180
CV104 ^**c**^	(TCCTG)_5_	GATTTTCGACTGTGCGCTTGG	AAGTTCTCTGCCGGAGCAAAC	2	64.0	F	6	133-158
CV106 ^**c**^	(GGC)_8_	AAACAGAGCATACTCGAGGCC	ACGTTGCTGACGAGGTTTTCC	2	64.0	F	6	146-161
CV115 ^**c**^	(TCACAG)_10_/(GTA)_6_/(AGA)_5_	GTCCATGTCCATCCATGATCC	GGAGCTCCGTCTTCTTGTTAC	2	60.0	S	6	150-174
CV119 ^**c**^	(AAAAC)_7_	TATCTCTCCTTGGTTTGGATGG	CACCCTACCAAATACCACAACA	2	64.0	F	5	121-175
CV128 ^**c**^	(GCA)_13_	AGGGCAACGGAGTCTTCGAC	CTGAACTCCGATGTGCTGGTG	2	60.0	F	5	147-168
CV135 ^**c**^	(AAG)_16_	AGCAAAACCAGCCTTCCCTTC	CTGTTTGTTTCTGCTTGCTTGC	2	64.0	F	6	129-159
CV144 ^**c**^	(TCTCCG)_5_	GCGCCTCCGTGGATAAGAATC	CCTTCCCCTACAGCGCCTAC	2	64.0	F	5	146-164

^a, b, c, d^ Develop by Oliveira *et al.*[72], Marconi *et al.*[73], CanaVialis (unpublished); Maccheroni *et al.*[74], respectively.

^e^ PCR program as described in Material and Methods.

^f^ Annealing temperature in the amplification reaction.

^g^ D: Silver-stained polyacrylamide gel electrophoresis (S) or fluorescence-based automated capillary electrophoresis (F) for the detection of EST-SSR alleles.

^h^ Observed number of alleles per locus and their size ranges in bp.

### Marker generation based on sugarcane retrotransposon sequences

**Figure 2:**
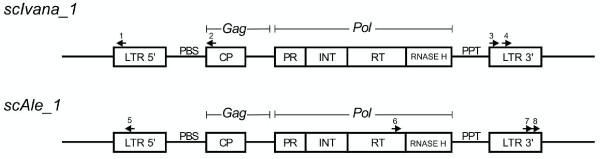
**Structure of sugarcane retrotransposons *scIvana_1* and *scAle_1.* ** Retrotransposons are LTR (long terminal repeats) consisting of elements within transcription initiation and termination sequences and detected as Gag, Pol, and Int domains that code for CP (capsid-like proteins), PR (protease), RT (reverse transcriptase), RNAase-H (ribonuclease H), and INT (integrase). Other sequences featured are PBS (primer binding sites), and PPT (polypurine tracts). Arrows indicate the primers designed for amplifying each of the elements, and synthesis direction. 1: *scIvana_1*-SSAP1; 2: *scIvana_1*-GagRev; 3: *scIvana_1*-LTR1; 4: *scIvana_1*-LTR2; 5: *scAle_1*-LTRr; 6: *scAle_1*-RT; 7: *scAle_1*-LTR1; 8: *scAle_1*-LTR2. Figures were not drawn to scale and were adapted from Kumar and Bennetzen [79].

The principle NBS-profiling technique [77] was applied according to Hanai *et al.*[78] to generate markers based on two retrotransposons named *scIvana_1* (GenBank Accession Number JN800016) and *scAle_1* (GenBank Accession Number JN800006). Approximately 500 ng of genomic DNA were digested with *Alu*I*, Dra*I, *Ssp*I, or *Rsa*I (NE BioLabs). Digestions were performed in a final volume of 30 μl using 7.5 U of enzyme for 7 h at 37°C, according to the manufacturer's recommendations. Reactions were terminated by heat inactivation (20 min at 65°C). Adapters were prepared by incubating equimolar amounts of LA (long arm) and SA (short arm) oligonucleotides at 65°C for 10 min, and respectively cooled to 37°C and 25°C (10 min each). The SA oligonucleotide 3’ end was blocked for *Taq* DNA polymerase extension by the addition of an amino group, but phosphorylated at the 5' end, which results in an adapter primer-annealing site only following the first PCR cycle. Subsequently, the digested material and a solution containing a 1.6 μM adapter (when restricted with *Alu*I or *Rsa*I), or a 0.2 μM adapter (when *Dra*I or *Ssp*I was used), 1 X ligation buffer (NE BioLabs), and 67 U T4 DNA ligase (400 units/μl; NE BioLabs) were mixed in equal volumes (30 μl), totaling 60 μl. Ligation was performed at 16°C for 16 h, and terminated by heat inactivation at 65°C for 20 min. The ligation products (diluted to 5 ng/μL) as template DNA were used to amplify selected fragments anchored to the retrotransposon sequence. A final volume 20 μl reaction mixture contained 4 μl of ligation products, 300 nM of each primer (a primer complementary to the adapter, and a primer complementary to the retrotransposon sequence, Table 5, Figure 2), 1.5 mM MgCl_2_, 0.2 mM each dNTP, 1 X buffer enzyme, and 2 U *Taq* DNA polymerase (Fermentas). PCR was conducted under the following conditions: an initial denaturation step at 94°C for 5 min; followed by 8 cycles at 94°C for 45 s, 58°C (− 1°C per cycle) during 50 s, and 72°C for 1 h:15 min; 25 cycles at 94°C for 45 s, 50°C for 50 s, and 72°C for 1 min; and a final extension at 72°C for 10 min. After PCR, the protocol for preparing AFLP gels was completed, followed by electrophoresis, and gel staining.

**Table 5:** Primer sequences used for the generation of sugarcane retrotransposons-based markers

**Primer**	**Sequence 5**′→**3**′ ^**a**^
Short arm oligonucleotide (AS)	TGGGATCTATACTT - H_2_N
Long arm oligonucleotide (LA)	ACTCGATTCTCAACCCGAAAGTATAGATCCCA
Adapter primer (AP)	ACTCGATTCTCAACCCGAAAG
*scIvana1*_SSAP1 (sIvSI)	CAAGCCCTTAATAGCAGAAA
*scIvana1*_GagRev (sIvGR)	TCCCTGTATACAACCCTGTC
*scIvana1*_LTR1 (sIvLI)	AGTCCTGCTCCCAGTTATCA
*scIvana1*_LTR2 (sIvLII)	GTCGCCTGGGTGTGTTATC
*scAle1*_LTRr (sAlLr)	ATACATGGGCCACATGGG
*scAle1*_RT (sAlRT)	CCTCCCDTCCTCGACCTTC
*scAle1*_LTR1 (sAlLI)	CCATGWGRCTAGGCCCATGTGGC
*scAle1*_LTR2 (sAlLI)	GGGGTGTTGGAGTGTGATTG

^a^ D = A, G or T; R = A or G; W = A or T.

Sixteen restriction enzyme and retrotransposon complementary primer combinations (for each sugarcane retrotransposon) were examined using the DNA from both parents in duplicate reactions. Combinations that exhibited clear band distribution over the gels, and revealed polymorphism rates ≥ 15% were selected for genotyping the F_1_ population.

### Marker nomenclature, genotyping and segregation analyses

The nomenclature adopted for AFLP primers followed the Keygene standard primer list [80] followed by the corresponding molecular size (in bp) of the band. A primer code was adopted for EST-SSR loci (ESTA, ESTB, ESTC and CV) followed by the molecular size of the allele (in bp). The retrotransposon-based locus nomenclature was an abbreviation that indicated the enzymes used in the digesting reaction (AI for *Alu*I, DI for *Dra*I, SI for *Ssp*I, and RI for *Rsa*I) followed by the primer code (Table 5), and the molecular size of the fragment (in bp). Wu’s [31] loci-segregation pattern notations follow all marker abbreviations e.g., D1-E35M47510 (Figure 1). We assumed the presence of a band, denoted by *A*, dominant over all *0* or null alleles (or simplex configuration with eight homo(eo)logous copies in the sugarcane genome), independent of marker type. Loci were denoted as “D1” when ‘IAC66-6’ was heterozygous for band presence, and the other parent was homozygous for band absence (*A0000000 x 00000000*). Loci were denoted as “D2” when ‘TUC71-7’ was heterozygous and ‘IAC66-6’ was homozygous. These loci were expected to segregate in a 1:1 fashion in the F_1_ population. “C” loci were heterozygous in both parents (*A0000000 x A0000000*), and exhibited a 3:1 segregation ratio. Differences between observed and expected proportions were compared using Chi-square test, assuming a polyploid model based on single-dose markers (SDM) for analyzing segregation in outcrossing species [14]. For minimizing problems caused by multiple comparisons, the Bonferroni correction was performed. Chi-square tests and Bonferroni adjustments for the effects of multiple comparisons were performed using the software R, v. 2.13.0 [81]. Loci with segregation distortion were not included in linkage analysis.

### Linkage analyses, map construction, and identification of sugarcane homo(eo)logous groups

All linkage analyses were performed using OneMap software [82]. Version 2.0-1 was preferred to construct a multipoint maximum likelihood linkage map applying a Hidden Markov Model approach [83].

Firstly, co-segregation groups (CGs) were established using a LOD score ≥ 6.0, and a recombination fraction ≤ 0.35. For groups with six or less markers, the best order was obtained by comparing all possible orders choosing the one with highest likelihood using the algorithm implemented in the command named “compare”. To obtain the best order for larger groups (more than six markers), the command “order.seq” was applied. In this case, the likelihood was the criteria used to place the markers along the CGs under a multipoint approach, as validated by Mollinari *et al.*[84]. Additionally, the “ripple” command was used to check for alternative orders, as well a visual inspection on the matrix containing the pairwise recombination fractions and LOD scores (heatmaps) for the CGs. The commands “compare”, “order.seq” and “ripple” were similar to those in the MAPMAKER/EXP software [17]. Finally, multipoint estimates of recombination fractions were calculated and converted into linkage distances using the Kosambi map function [85]. Map drawings were generated using MapChart 2.2 [86].

Due to the multiallelic nature and known polymorphisms, EST-SSR loci are valuable in recognizing homo(eo)logous groups (HGs) in sugarcane. Initially, CGs were assigned to HGs if they contained at least two of the same EST-SSRs. In addition, CGs were putatively added if they contained an EST-SSR locus in common with the HG [28,30,32,54]. Using this practice, two HGs were established, I and IV. Then due to the insufficient amount of SSRs, in a number of instances only one locus was used to assign CGs to the following putative HGs, II, III, V, VI and VII [30,32]. We applied Roman numerals to denote HGs; within each HG, CGs were classified in a descending order according to size (in cM). The unassigned groups were designated U, and also classified according to their size.

Finally, to test if *scIvana_1*-based markers have a tendency to be clustered along the genome, we used an approach similar to the one presented by Echt *et al.*[87]. The genetic map was divided in 10 cM bins and the number of *scIvana_1*-based markers in each interval was recorded. If markers were randomly distributed, they would follow a Poisson distribution [47], defined as P(x) = λ^x^e^-λ^/x!, where P(x) is the probability function; x is the number of markers observed in the intervals (ranging from 0 to 3), λ is the distribution parameter calculated as average number of markers per interval in the map. A Chi-square goodness-of-fit test was performed, with 2 degrees of freedom (df = c −1- r, where c is the number of classes and r is the number of estimated parameters, r = 1).

### Molecular validation of *scIvana_1*-based fragments

Verification that amplified DNA fragments were derived from retrotransposon templates was conducted by excising DNA fragments from polyacrylamide gels, eluting DNA in a TE solution (10:1), and re-amplifying the DNA fragments. Five μl of the diluted DNA mixture was added to 50 μL of the same solution used for retrotransposon-based marker generation, however primer concentration was 30 nM, and *Taq* DNA polymerase was 5 U. The PCR program was simplified and conducted under the following conditions: an initial denaturation step at 94°C for 5 min; 30 cycles of 94°C for 30 s, 55°C for 30 s; 72°C for 42 s; and a final extension at 72°C for 10 min. For sequencing, PCR fragments were resolved on agarose gels, purified with the QIAEX II Gel Extraction kit (QIAGEN), and cloned into pMOS Blue Blunt-Ended Cloning Kit (GE Healthcare Life Sciences). Inserts were sequenced in the forward direction. Sequencing reactions were performed according to Sanger *et al.*[88] using DYEnamicTM ET Dye Terminator Cycle Sequencing Kit (Amersham Pharmacia Biotech, Inc.) on an ABI 3730 system (Applied Biosystems). Sequence quality was examined using the Phred/Phrap/Consed package [89]. Nucleotide sequences were compared to reference data available at Genbank by BLAST analysis [90].

## Abbreviations

AFLP, Amplified Fragment Length Polymorphism; BC, Backcross; CG, Co-segregation Group; DH, Double Haploid; EST, Expressed Sequence Tag; EST-RFLP, Expressed Sequence Tag-Restriction Fragment Length Polymorphism; EST-SSR, Expressed Sequence Tag-Simple Sequence Repeat; HG, Homo(eo)logous group; IRAP, Inter-Retrotransposon Amplified Polymorphism; ISBP, Insertion Site-Based Polymorphism; LG, Linkage Group; LRR, Leucine Rich Repeat; NBS-LRR, Nucleotide Binding Site–Leucine Rich Repeat; QTL, Quantitative Trait Loci; RBIP, Retrotransposon-Based Insertion Polymorphism; REMAP, Retrotransposon Microsatellite Amplified Polymorphism; RFLP, Restriction Fragment Length Polymorphism; RIL, Recombinant Inbreed Line; SDM, Single Dose Marker; SNP, Single Nucleotide Polymorphism; SSAP, Sequence Specific Amplified Polymorphism; SSR, Simple Sequence Repeat (or Microsatellite); S-T Kinase, Serine-Threonine Kinase; SUCEST, Sugarcane Expressed Sequence Tag; TE, Transposable Element; TRAP, Target Region Amplification Polymorphism.

## Competing interests

The authors declare that they have no competing interests.

## Authors’ contributions

ACP and TBRM ran AFLP, SSR and *scIvana_1*-based markers, created the figures and tables, and drafted the manuscript; MM and RG constructed the linkage map, and applied all statistical tests cited under and the supervision of AAFG; and MAVS and DSD described sugarcane *scIvana_1* and *scAle_1* retrotransposons. MLCV conceived the study, and wrote the final manuscript. APS and TM developed some of the SSRs used here. WMJr and HJJr provided the plant material as well as developed most of the SSRs. All authors read and approved the manuscript.

## Supplementary Material

Additional file 1 **Amplification patterns obtained from AFLP, EST-SSR, and *scIvana_1* -based markers for the sugarcane mapping population.** Several segregating alleles are shown, as well as molecular weight standard (lane M) fragment sizes. Codes correspond to parental and F_1_-progeny genotypes. Click here for file

Additional file 2 **Genetic information provided by the AFLP, EST-SSR and the retrotransposon *scIvana_1-* based loci.** Click here for file
