# Supplementary figures and images for: A novel linkage map of sugarcane with evidence for clustering of retrotransposon-based markers
Source: BMC Genet. 2012 Jun 28;13:51. doi: 10.1186/1471-2156-13-51 (PMC3443450; doi:10.1186/1471-2156-13-51)

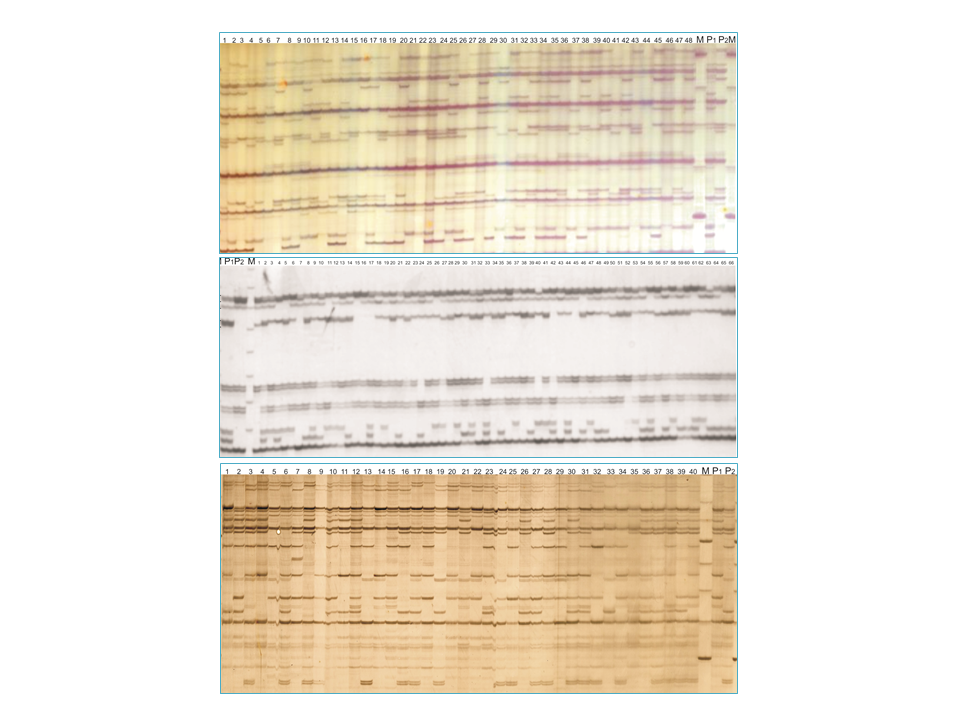

Supplement: Additional file 1 — Amplification patterns obtained from AFLP, EST-SSR, and scIvana_1 -based markers for the sugarcane mapping population. Several segregating alleles are shown, as well as molecular weight standard (lane M) fragment sizes. Codes correspond to parental and F1-progeny genotypes. [file 1471-2156-13-51-S1.tiff]
